# Supplementary figures and images for: Inhibition of β1-AR/Gαs signaling promotes cardiomyocyte proliferation in juvenile mice through activation of RhoA-YAP axis
Source: eLife. 2022 Dec 8;11:e74576. doi: 10.7554/eLife.74576 (PMC9767473; doi:10.7554/eLife.74576)

Figure4 figure supplement-2-source data

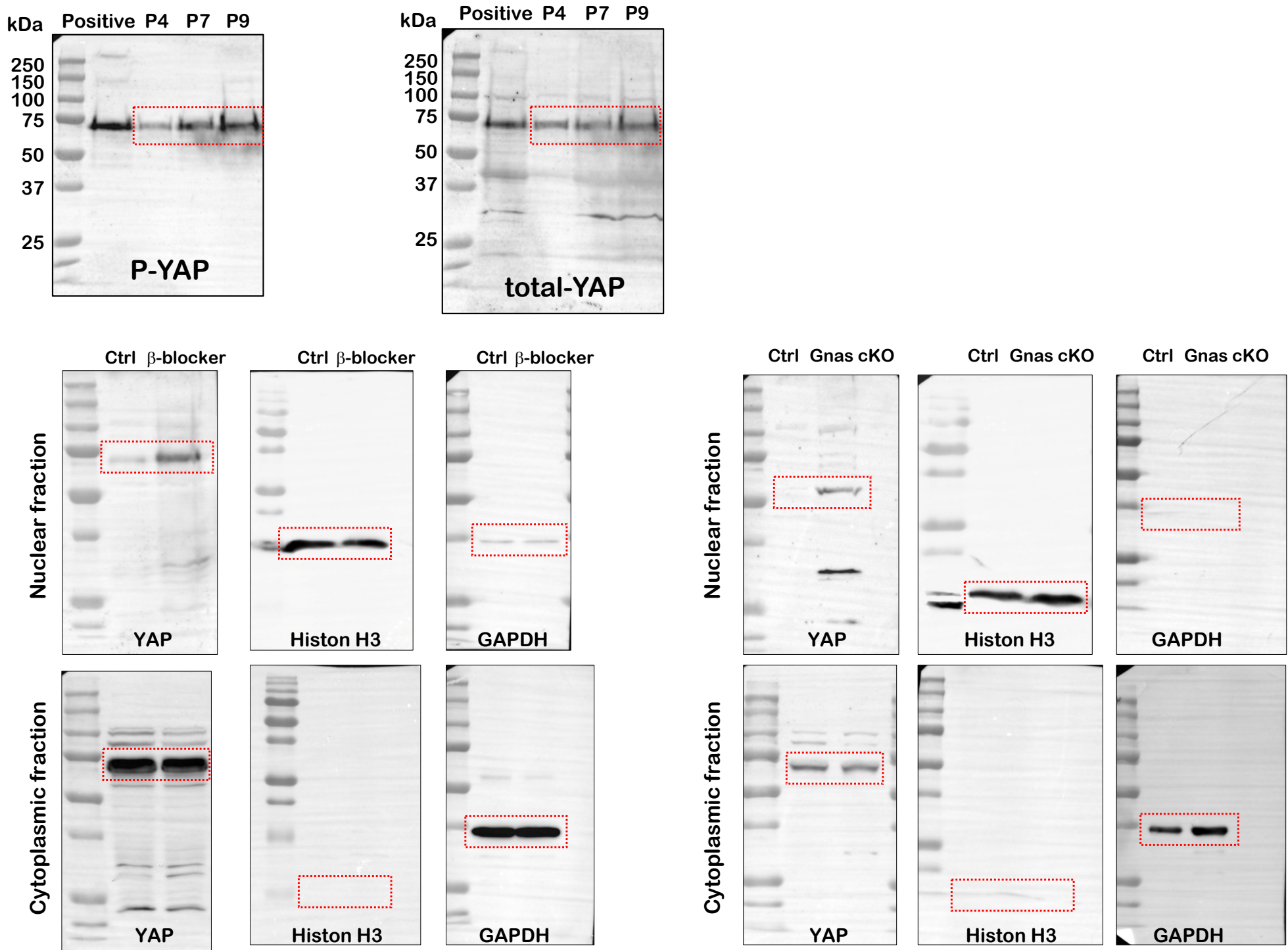

Supplement: Figure 4—figure supplement 2—source data 1. [file elife-74576-fig4-figsupp2-data1.zip › Source data Figure4-figure supplement2/Figure4-figure supplement-2-source data.pdf]

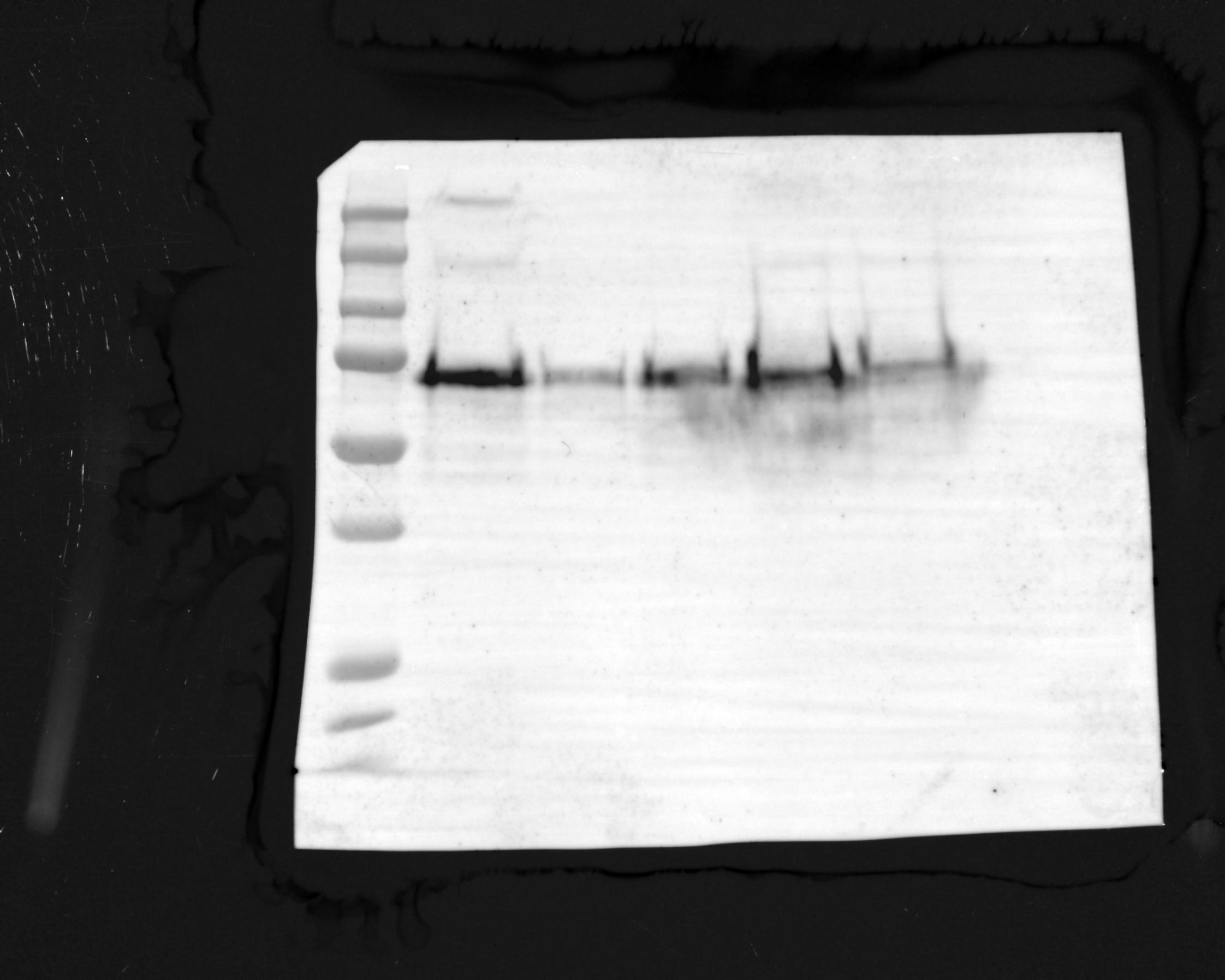

Supplement: Figure 4—figure supplement 2—source data 1. [file elife-74576-fig4-figsupp2-data1.zip › Source data Figure4-figure supplement2/Figure4-figure supplement2 source data1.tif]

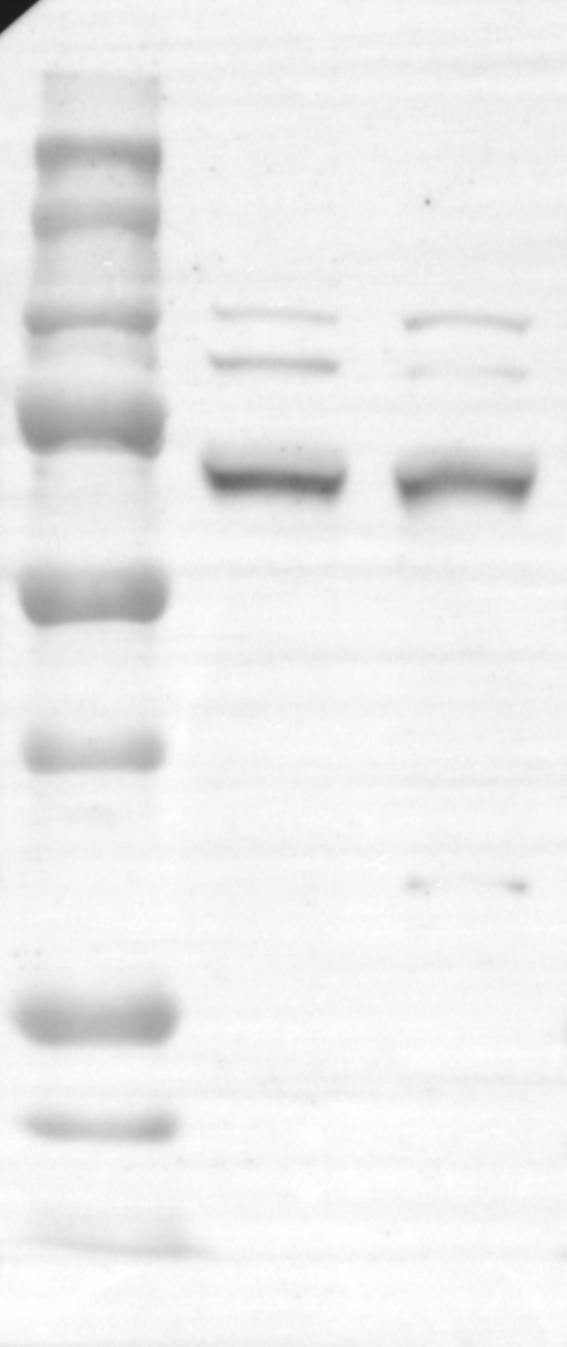

Supplement: Figure 4—figure supplement 2—source data 1. [file elife-74576-fig4-figsupp2-data1.zip › Source data Figure4-figure supplement2/Figure4-figure supplement2 source data10.tif]

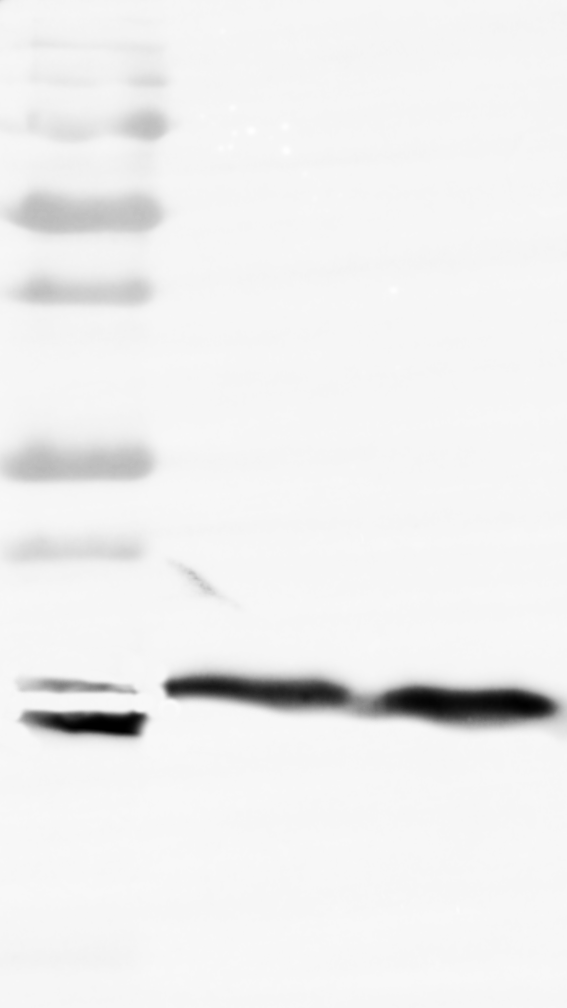

Supplement: Figure 4—figure supplement 2—source data 1. [file elife-74576-fig4-figsupp2-data1.zip › Source data Figure4-figure supplement2/Figure4-figure supplement2 source data11.tif]

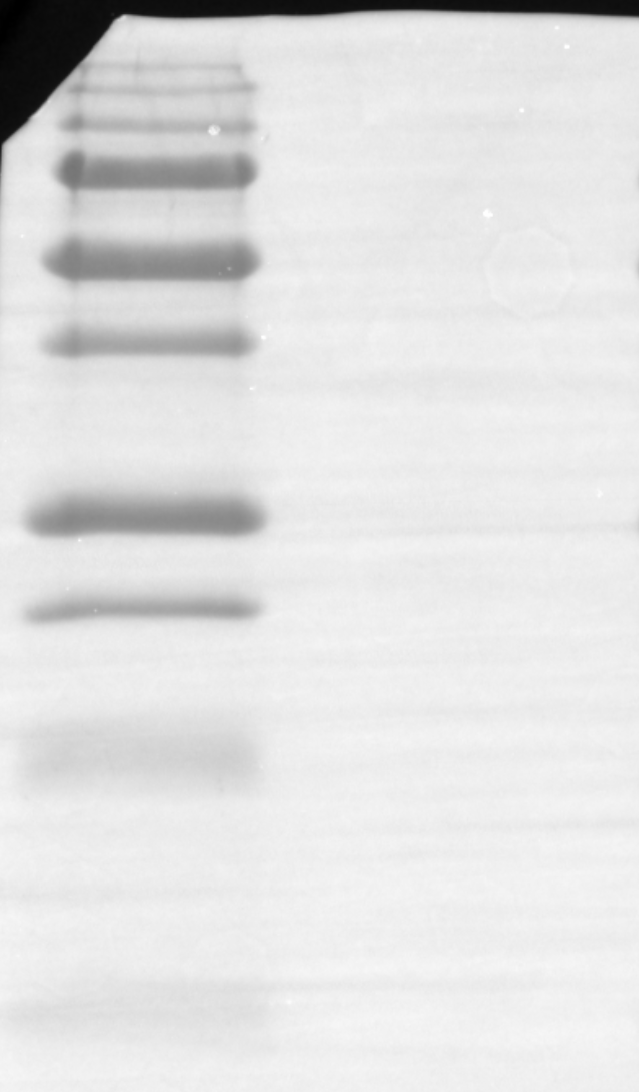

Supplement: Figure 4—figure supplement 2—source data 1. [file elife-74576-fig4-figsupp2-data1.zip › Source data Figure4-figure supplement2/Figure4-figure supplement2 source data12.tif]

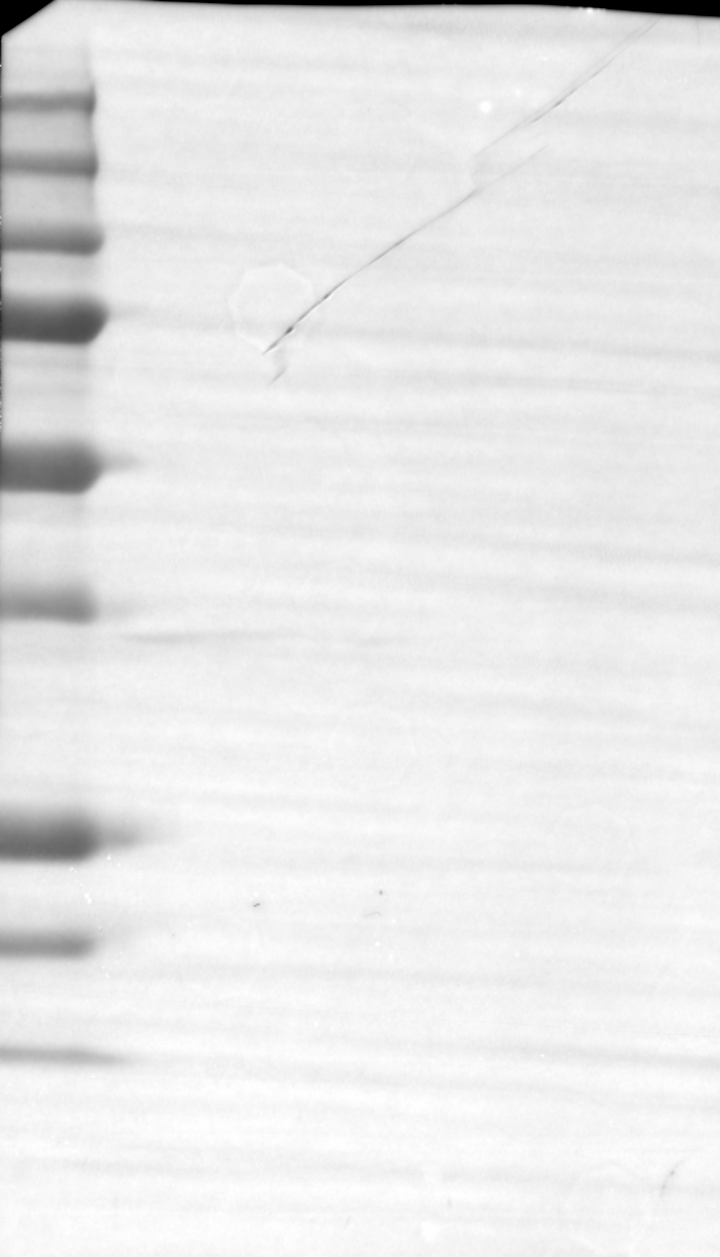

Supplement: Figure 4—figure supplement 2—source data 1. [file elife-74576-fig4-figsupp2-data1.zip › Source data Figure4-figure supplement2/Figure4-figure supplement2 source data13.tif]

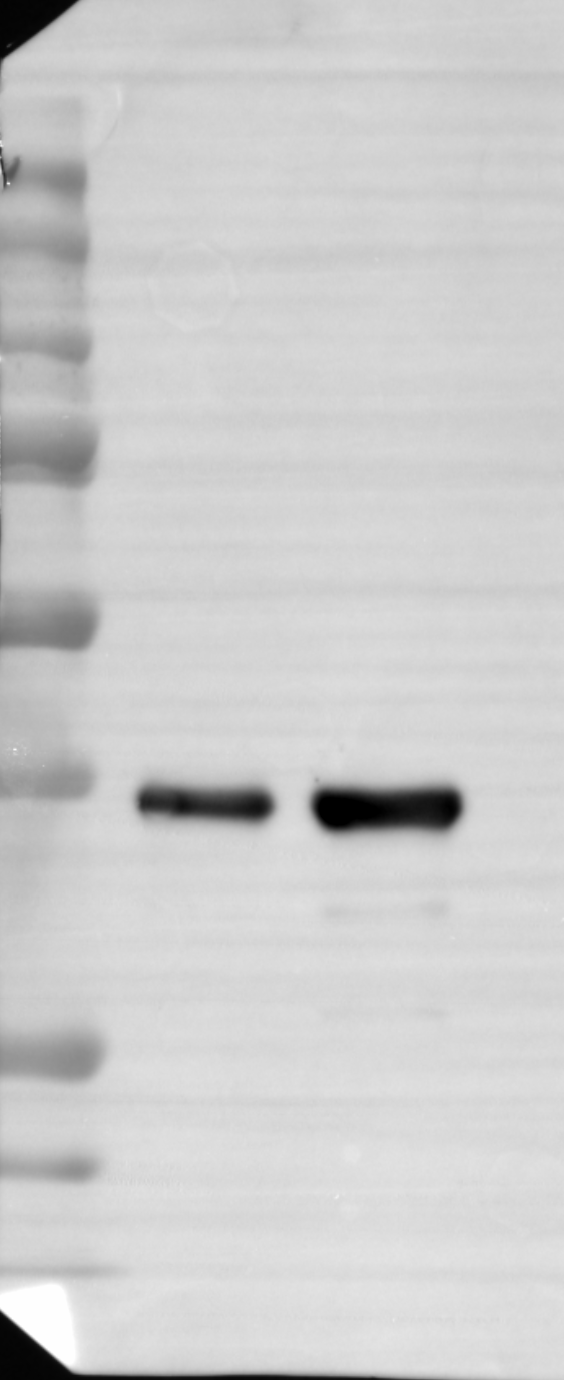

Supplement: Figure 4—figure supplement 2—source data 1. [file elife-74576-fig4-figsupp2-data1.zip › Source data Figure4-figure supplement2/Figure4-figure supplement2 source data14.tif]

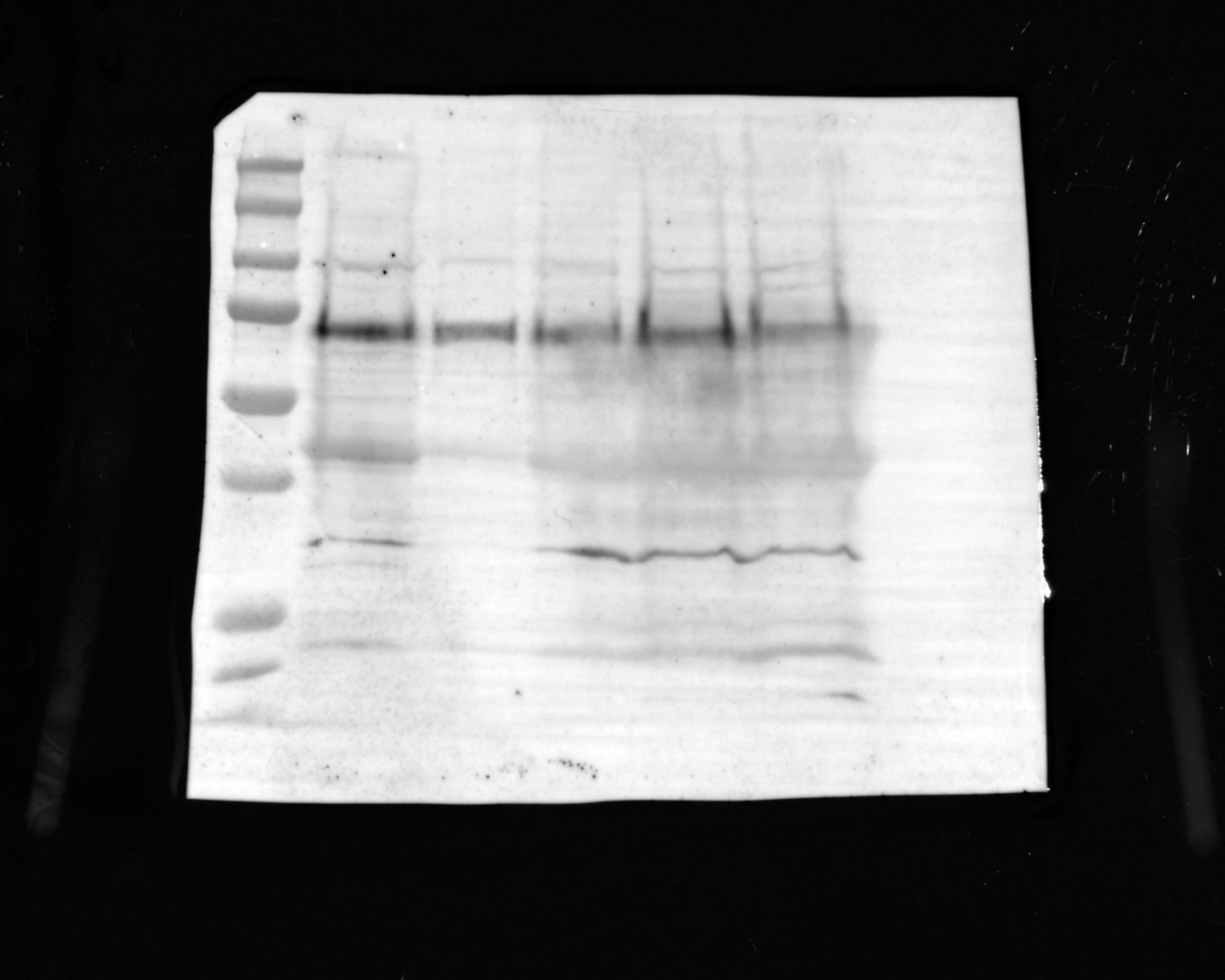

Supplement: Figure 4—figure supplement 2—source data 1. [file elife-74576-fig4-figsupp2-data1.zip › Source data Figure4-figure supplement2/Figure4-figure supplement2 source data2.tif]

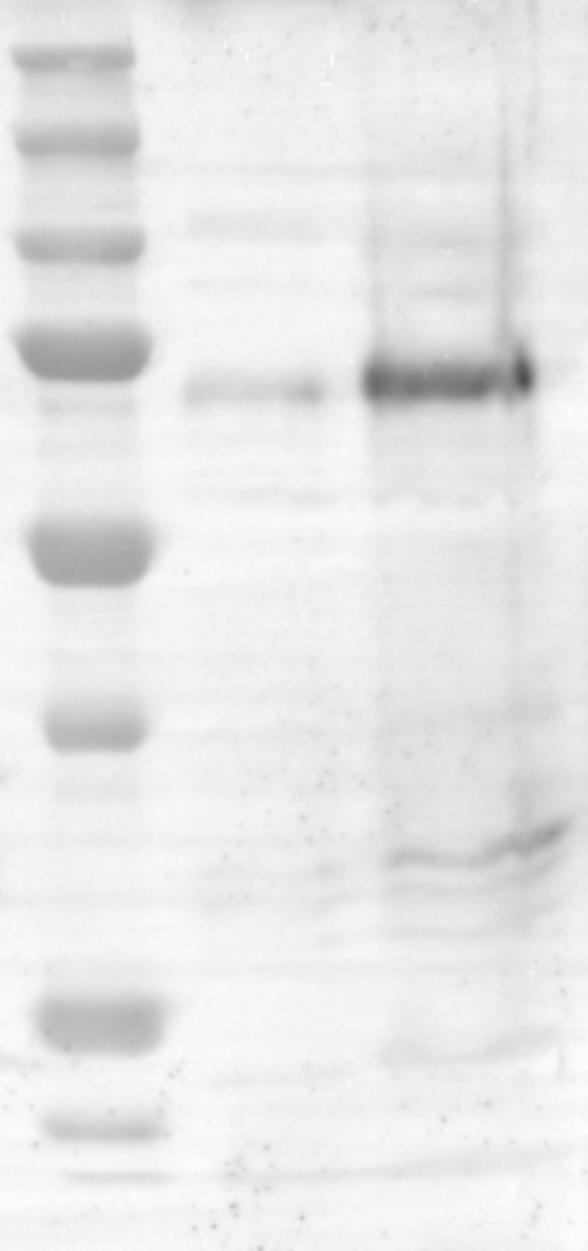

Supplement: Figure 4—figure supplement 2—source data 1. [file elife-74576-fig4-figsupp2-data1.zip › Source data Figure4-figure supplement2/Figure4-figure supplement2 source data3.tif]

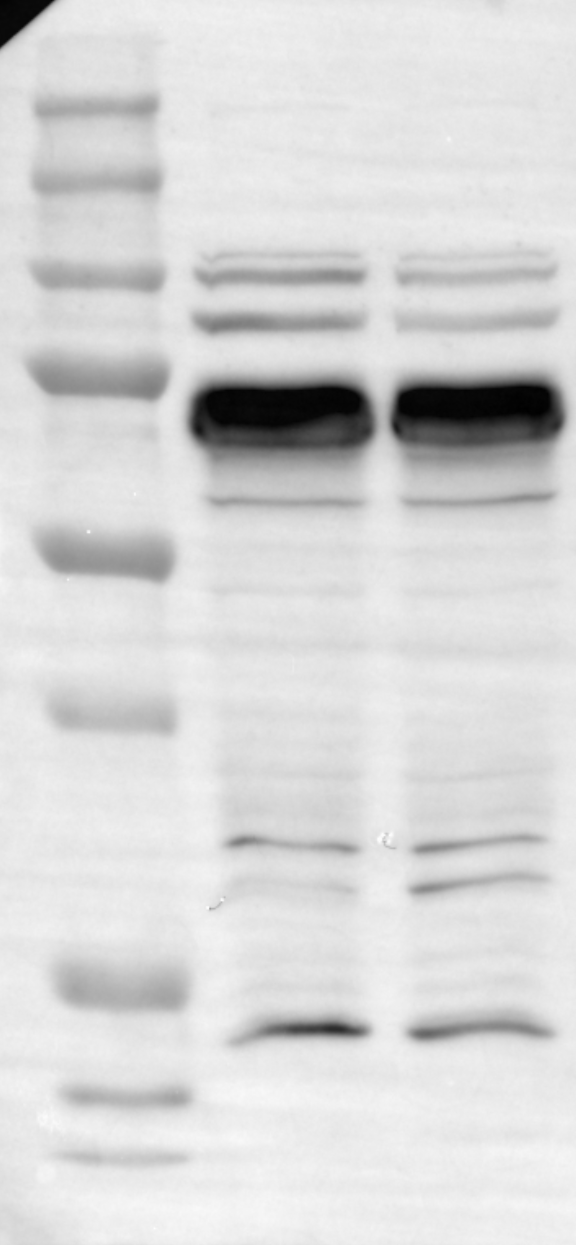

Supplement: Figure 4—figure supplement 2—source data 1. [file elife-74576-fig4-figsupp2-data1.zip › Source data Figure4-figure supplement2/Figure4-figure supplement2 source data4.tif]

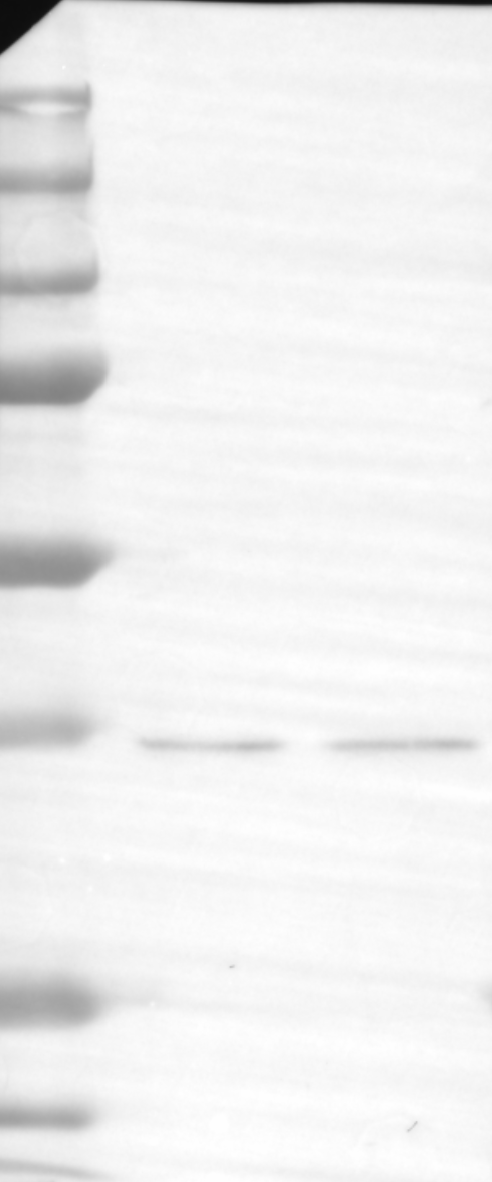

Supplement: Figure 4—figure supplement 2—source data 1. [file elife-74576-fig4-figsupp2-data1.zip › Source data Figure4-figure supplement2/Figure4-figure supplement2 source data5.tif]

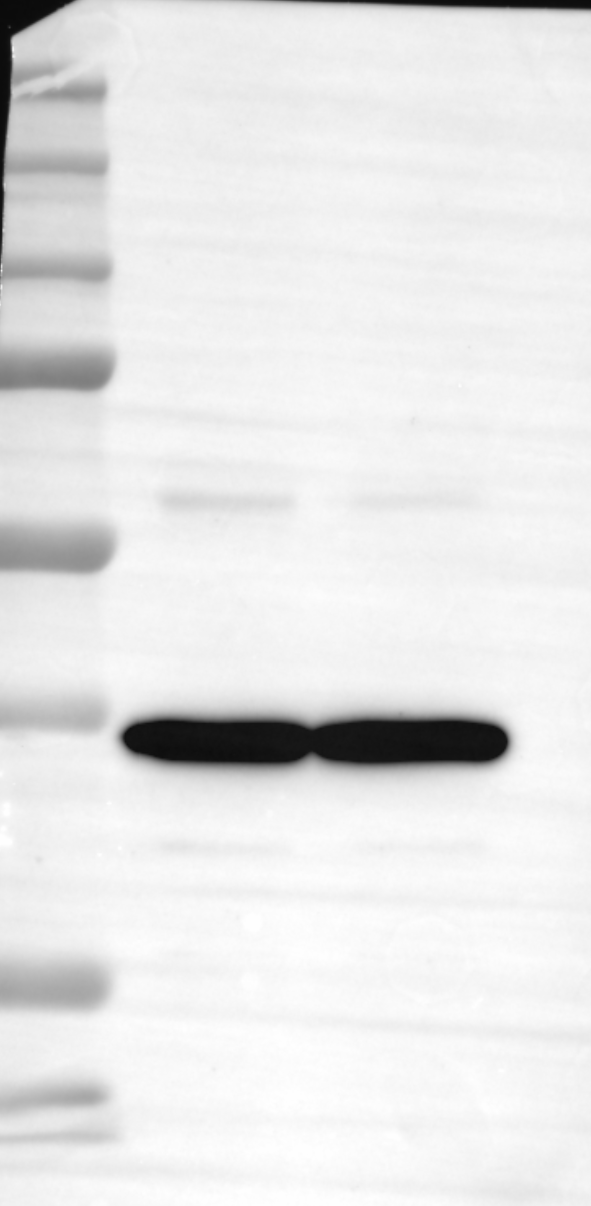

Supplement: Figure 4—figure supplement 2—source data 1. [file elife-74576-fig4-figsupp2-data1.zip › Source data Figure4-figure supplement2/Figure4-figure supplement2 source data6.tif]

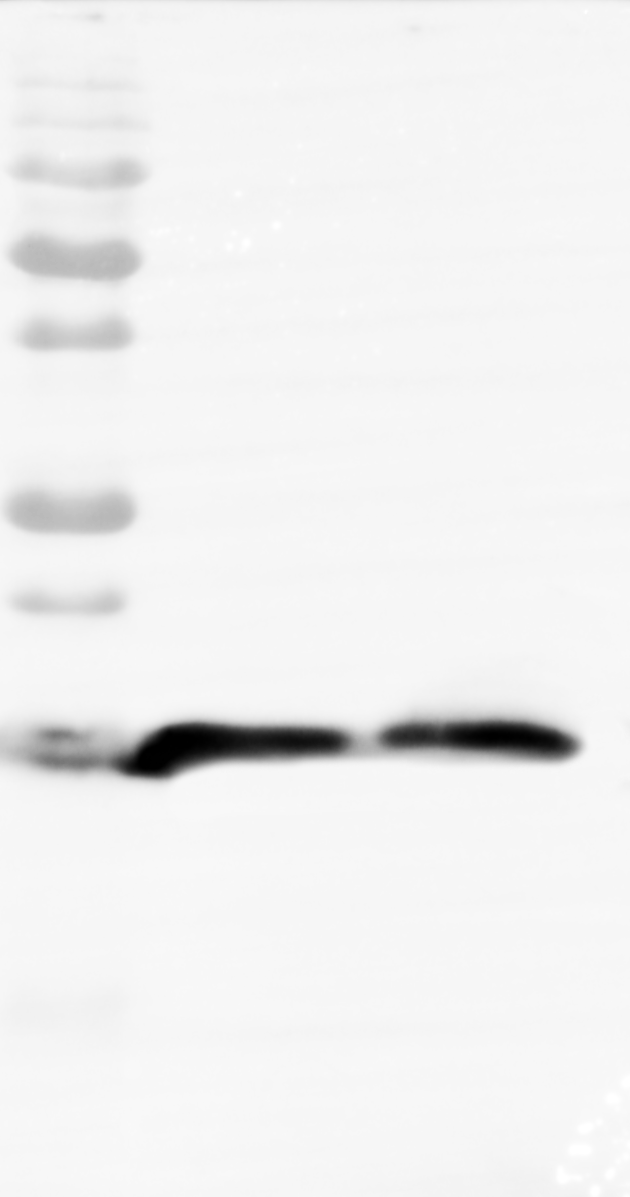

Supplement: Figure 4—figure supplement 2—source data 1. [file elife-74576-fig4-figsupp2-data1.zip › Source data Figure4-figure supplement2/Figure4-figure supplement2 source data7.tif]

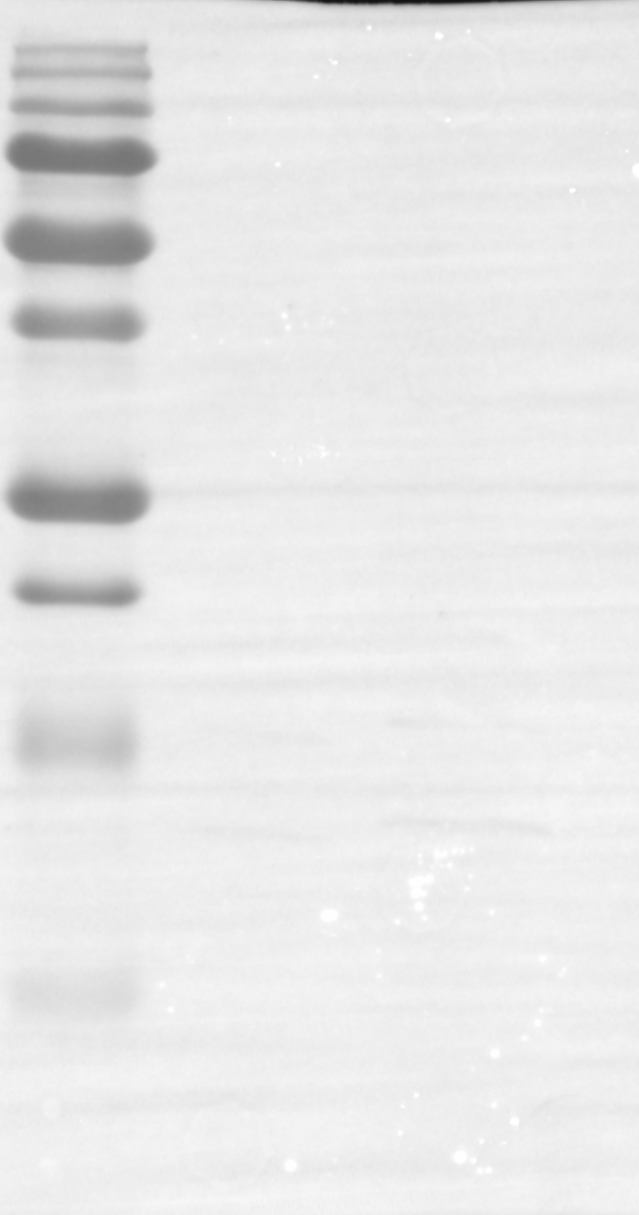

Supplement: Figure 4—figure supplement 2—source data 1. [file elife-74576-fig4-figsupp2-data1.zip › Source data Figure4-figure supplement2/Figure4-figure supplement2 source data8.tif]

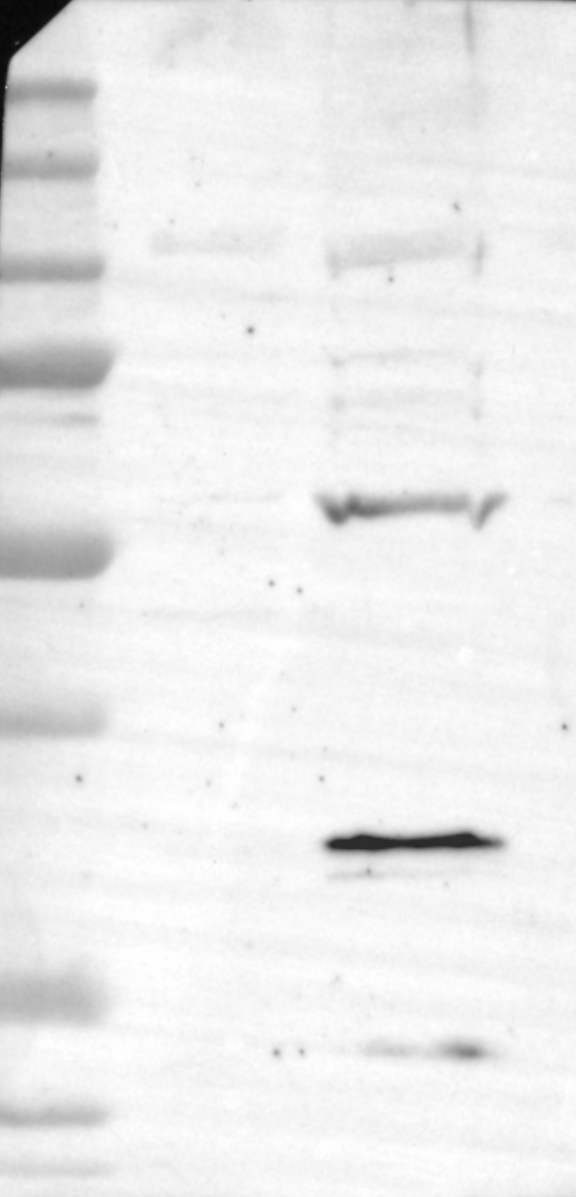

Supplement: Figure 4—figure supplement 2—source data 1. [file elife-74576-fig4-figsupp2-data1.zip › Source data Figure4-figure supplement2/Figure4-figure supplement2 source data9.tif]

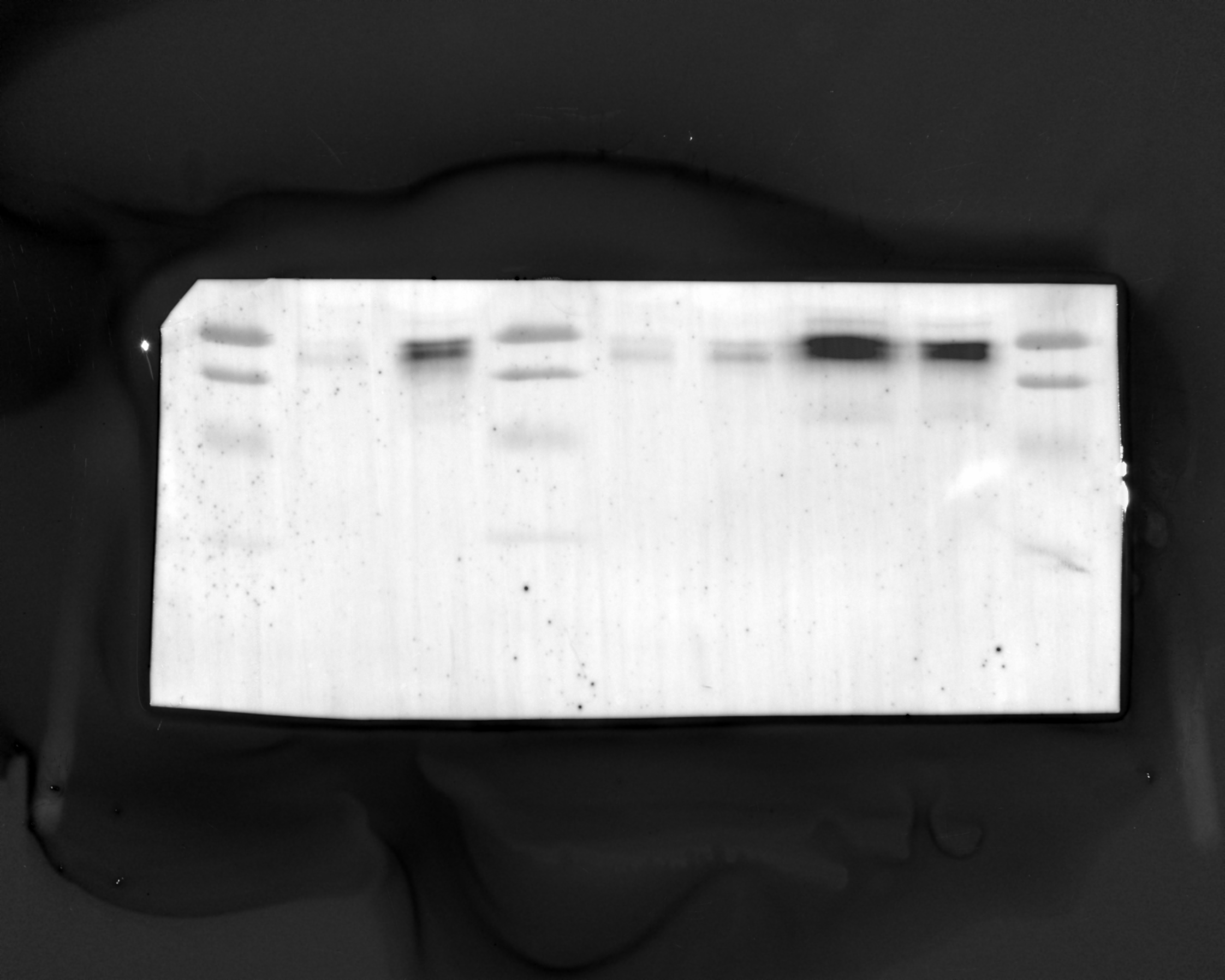

Supplement: Figure 5—source data 1. [file elife-74576-fig5-data1.zip › Source data Figure5/Figure5b-source data1 Total-RhoA.tif]

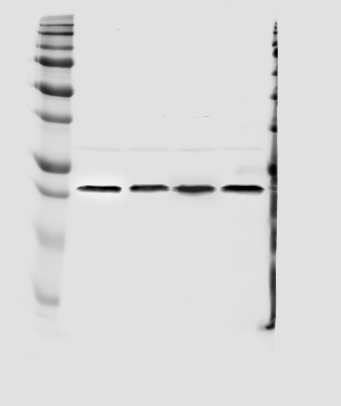

Supplement: Figure 5—source data 1. [file elife-74576-fig5-data1.zip › Source data Figure5/Figure5b-source data2 Total-RhoA.tif]

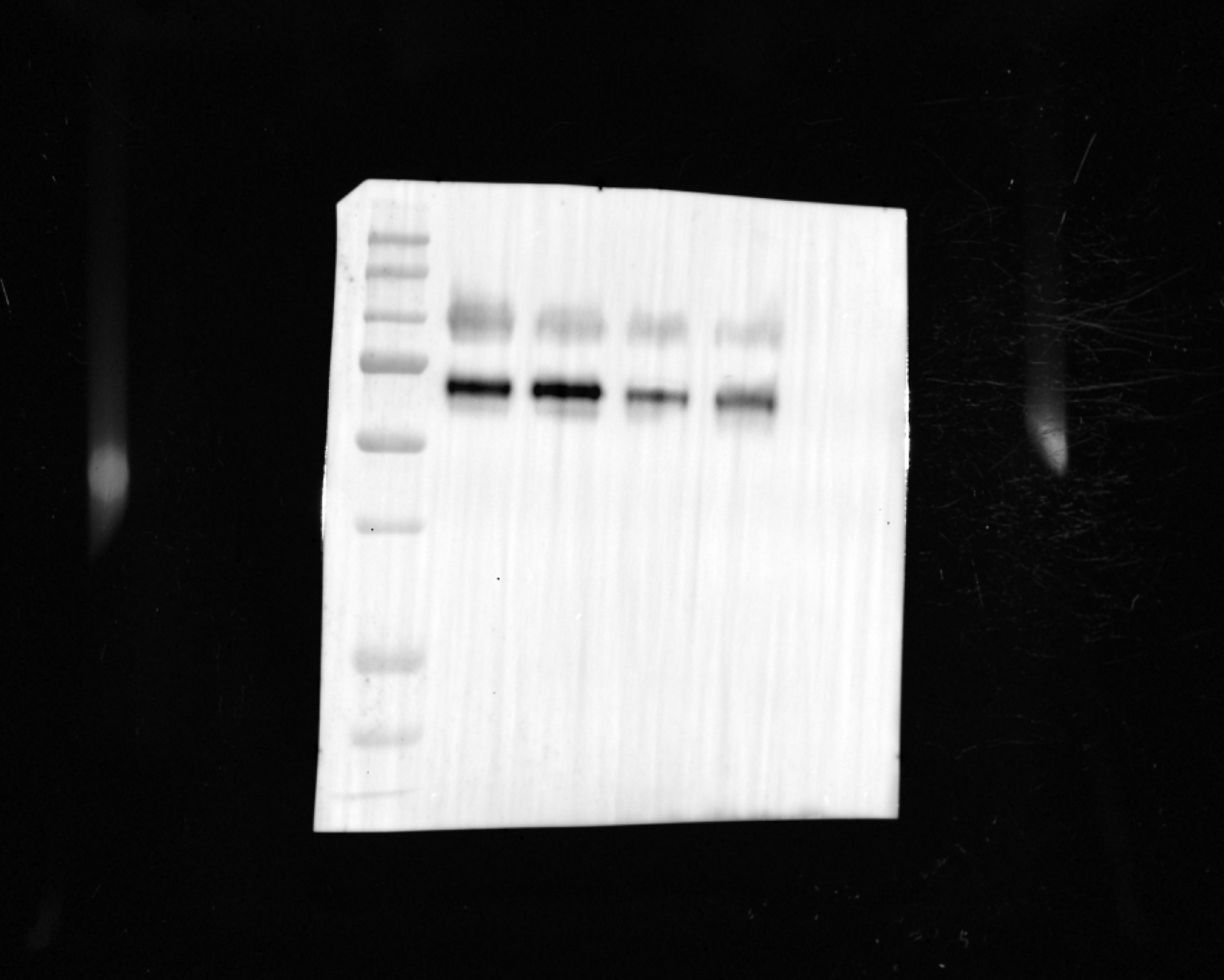

Supplement: Figure 5—source data 1. [file elife-74576-fig5-data1.zip › Source data Figure5/Figure5b-source data3 P-YAP.tif]

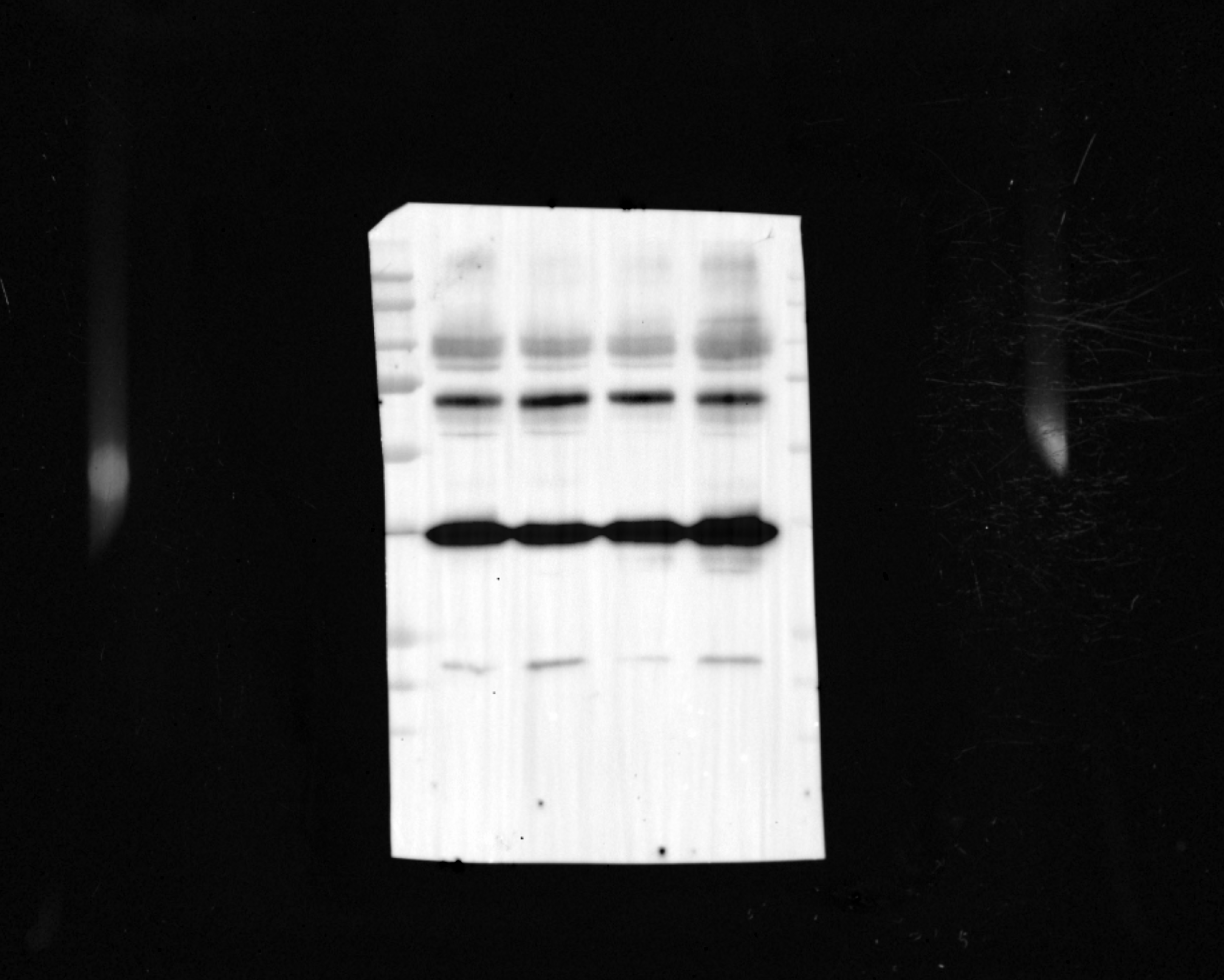

Supplement: Figure 5—source data 1. [file elife-74576-fig5-data1.zip › Source data Figure5/Figure5b-source data4 Total YAP.tif]

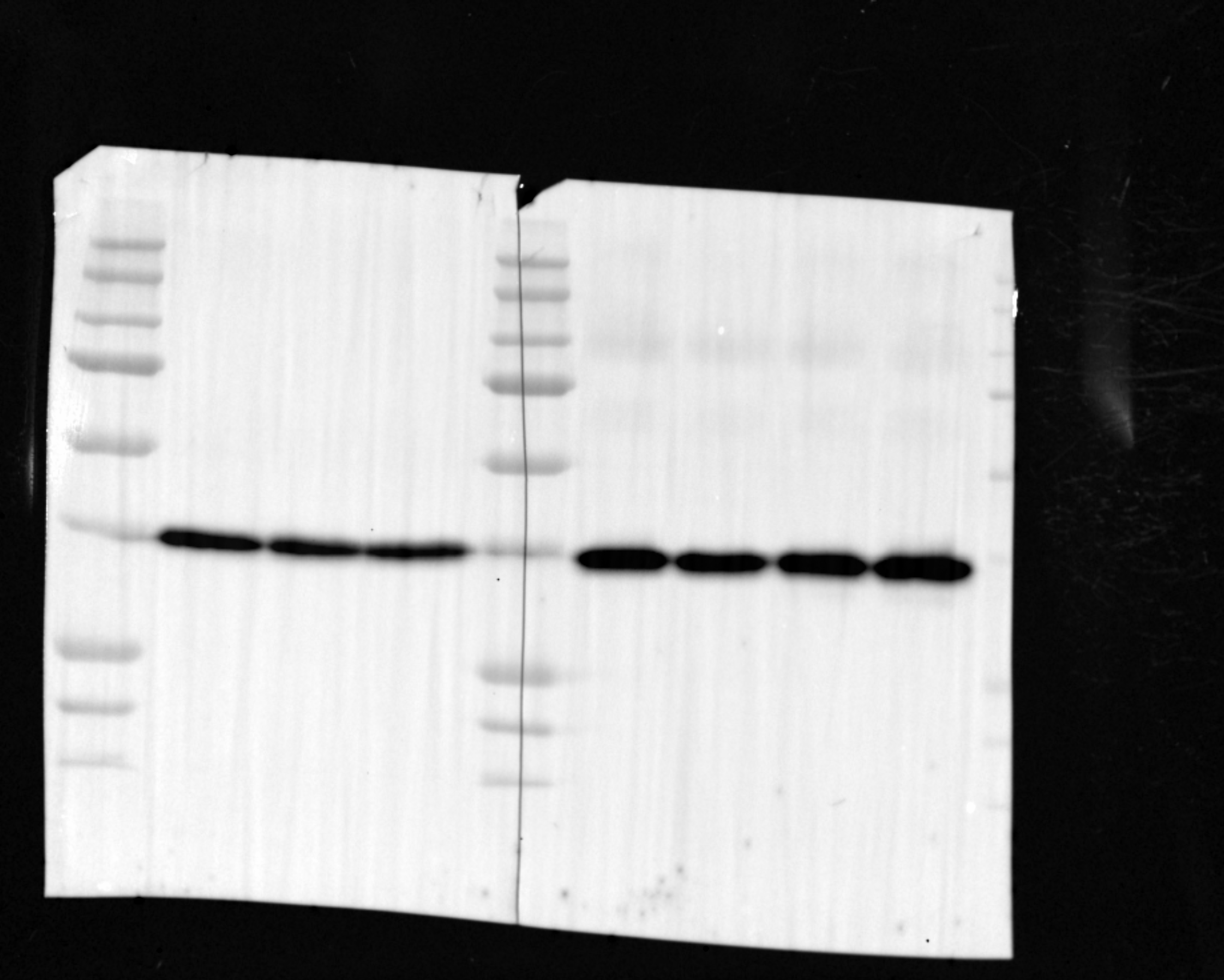

Supplement: Figure 5—source data 1. [file elife-74576-fig5-data1.zip › Source data Figure5/Figure5b-source data5 GAPDH (right).tif]

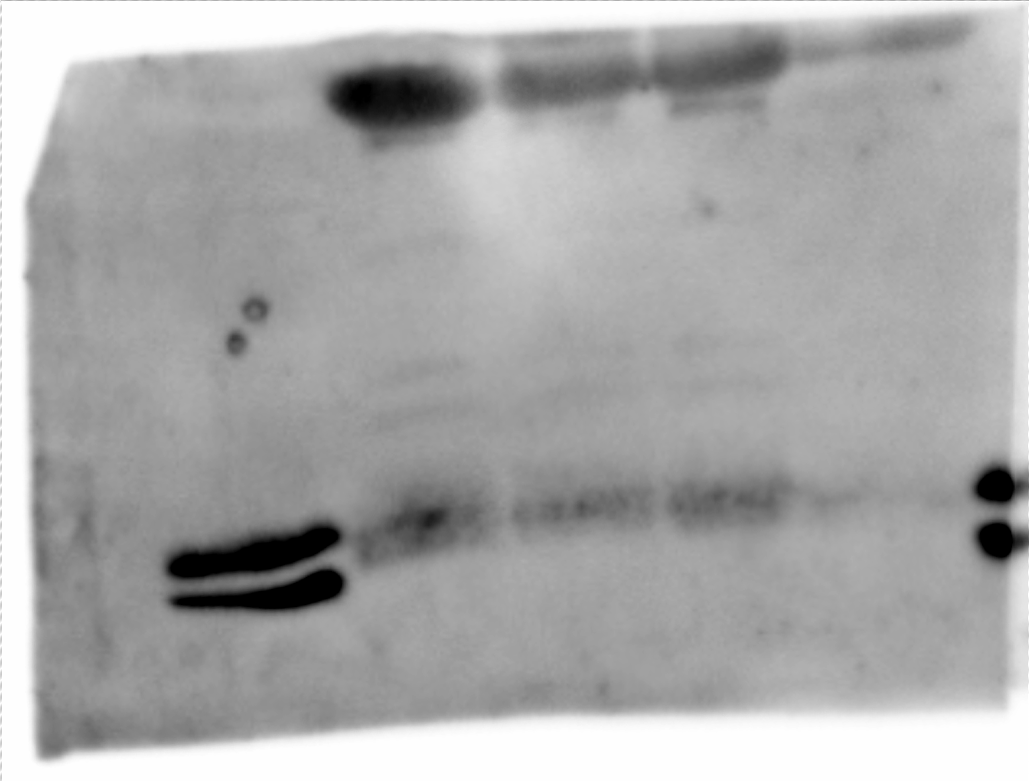

Supplement: Figure 5—source data 1. [file elife-74576-fig5-data1.zip › Source data Figure5/Figure5c-source data6 Active-RhoA.tif]

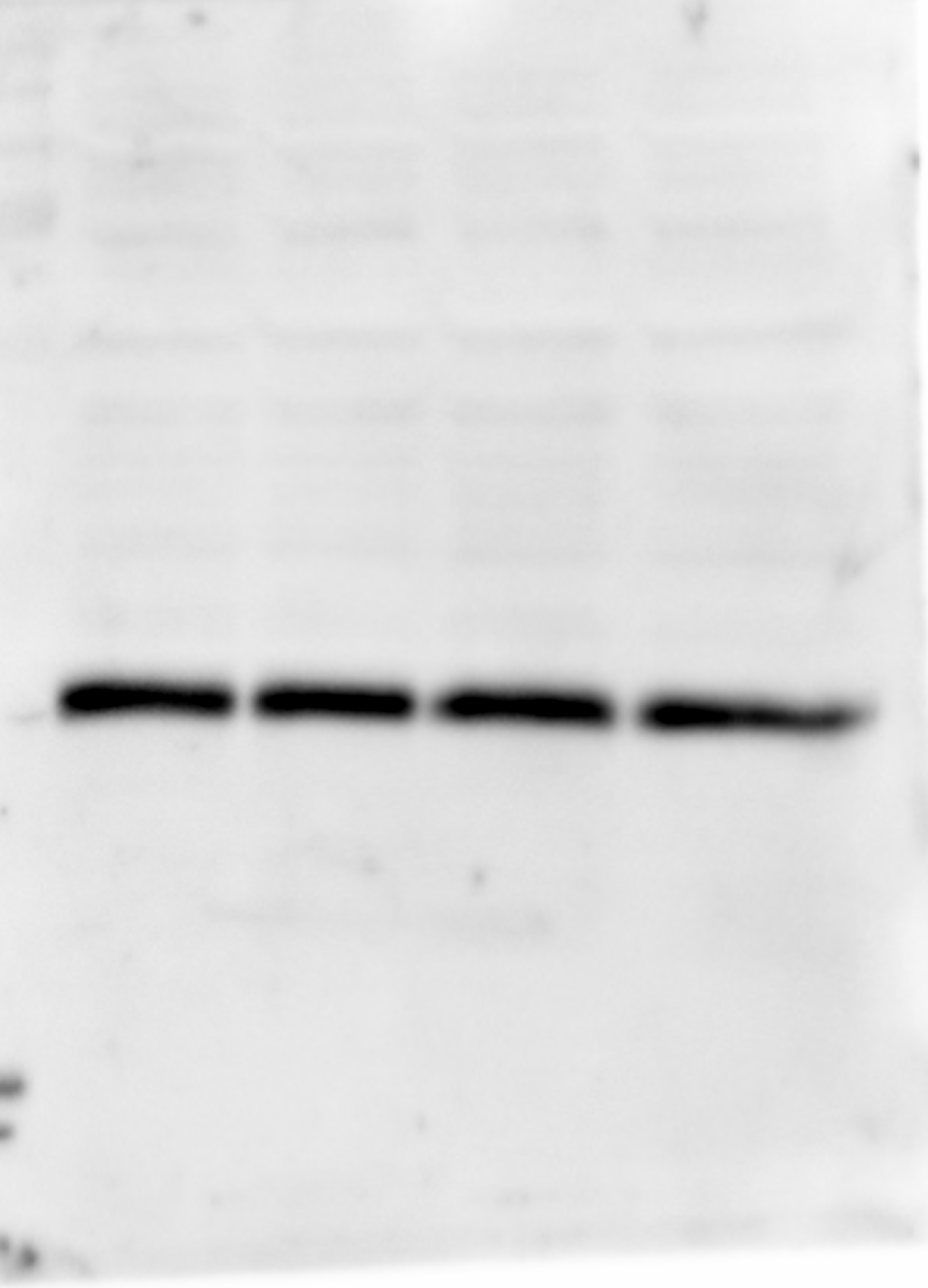

Supplement: Figure 5—source data 1. [file elife-74576-fig5-data1.zip › Source data Figure5/Figure5c-source data7 total RhoA.tif]

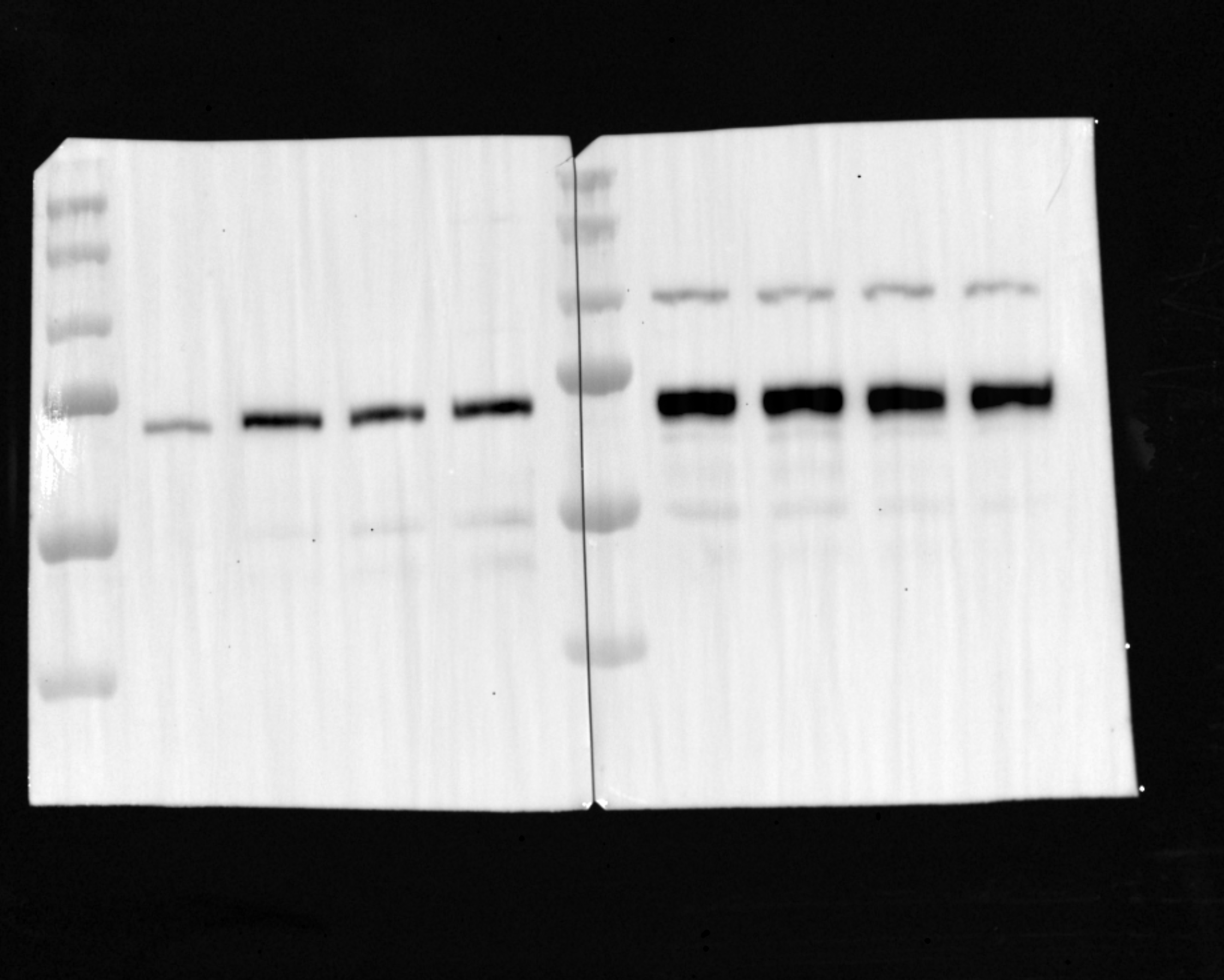

Supplement: Figure 5—source data 1. [file elife-74576-fig5-data1.zip › Source data Figure5/Figure5c-source data8 Active-RhoA (left) and total RhoA (right).tif]

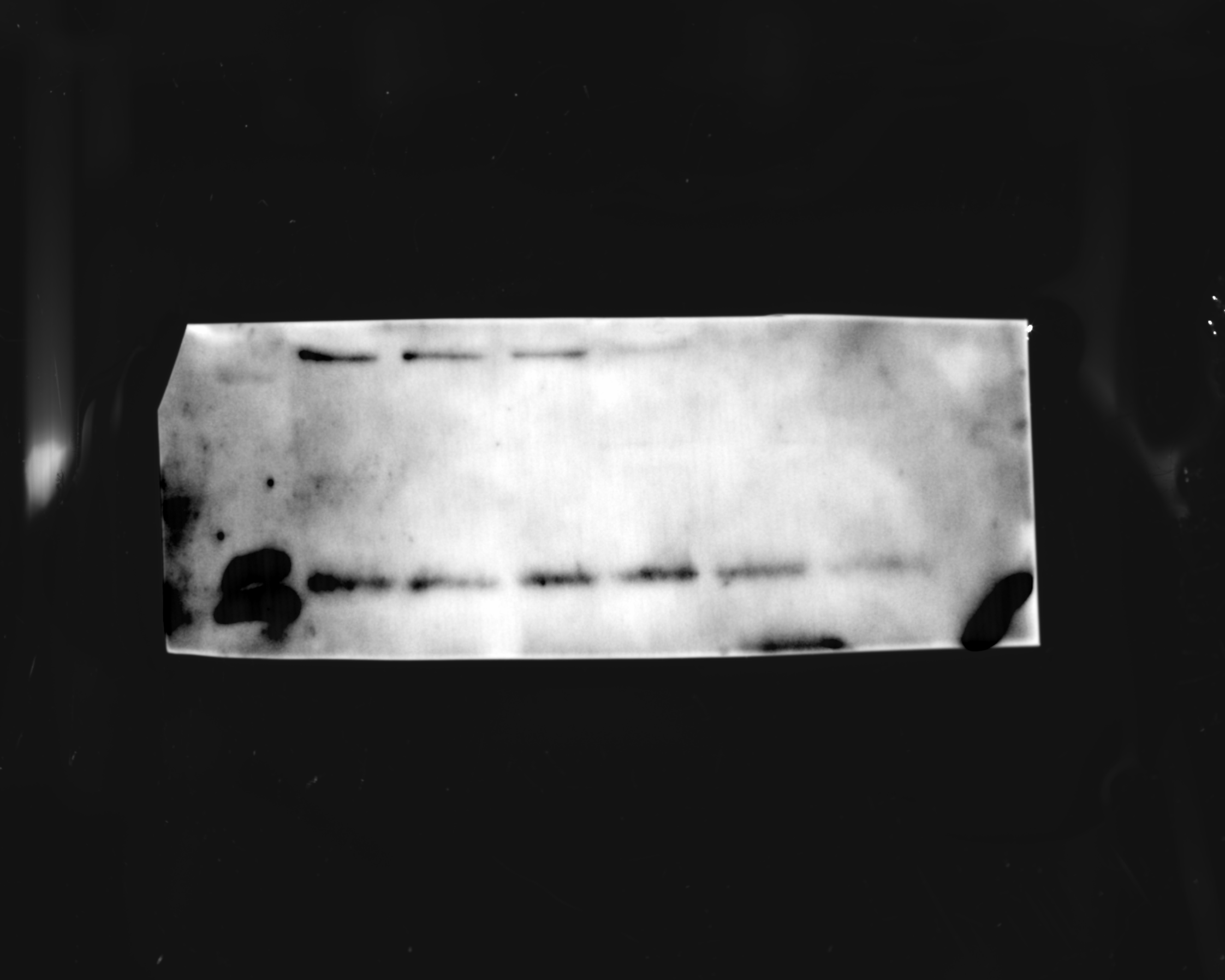

Supplement: Figure 5—figure supplement 1—source data 1. [file elife-74576-fig5-figsupp1-data1.zip › Source data Figure5-figure supplement1/Figure5-figure supplement1-source data1 Active-RhoA.tif]

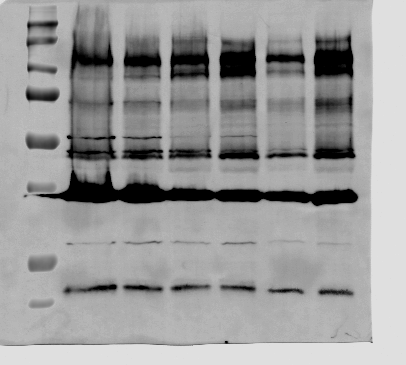

Supplement: Figure 5—figure supplement 1—source data 1. [file elife-74576-fig5-figsupp1-data1.zip › Source data Figure5-figure supplement1/Figure5-figure supplement1-source data2 Total RhoA.tif]
