# Supplementary material for: Inhibition of β1-AR/Gαs signaling promotes cardiomyocyte proliferation in juvenile mice through activation of RhoA-YAP axis
Source: eLife. 2022 Dec 8;11:e74576. doi: 10.7554/eLife.74576 (PMC9767473; doi:10.7554/eLife.74576)

Figure5b-source data1

Active RhoA

P7 Heart

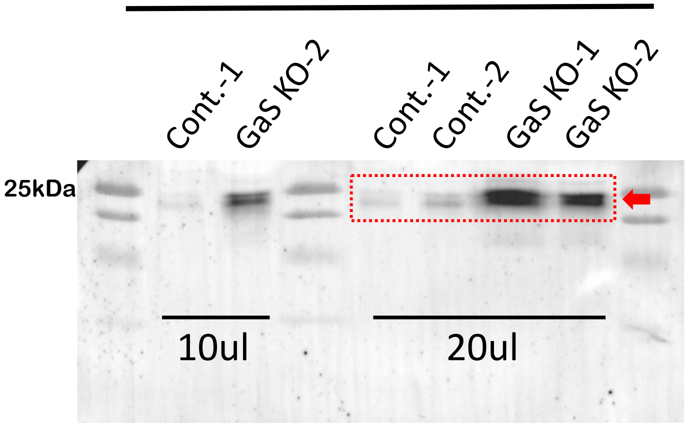

IB: RhoA

RhoA Ab.: CST Rabbit RhoA (2117S)

Figure5b-source data2

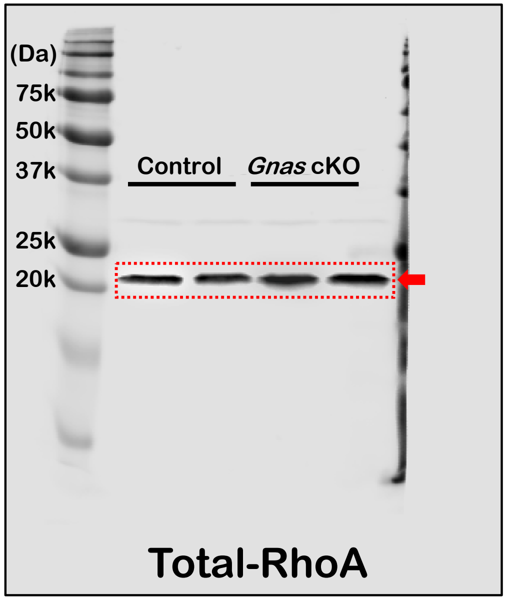

Figure5b-source data3

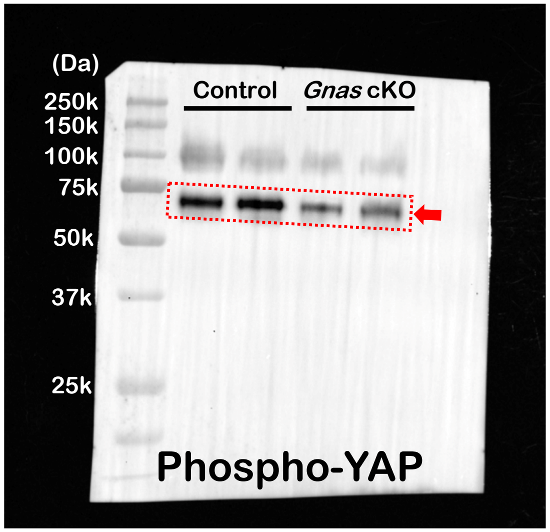

Figure5b-source data4

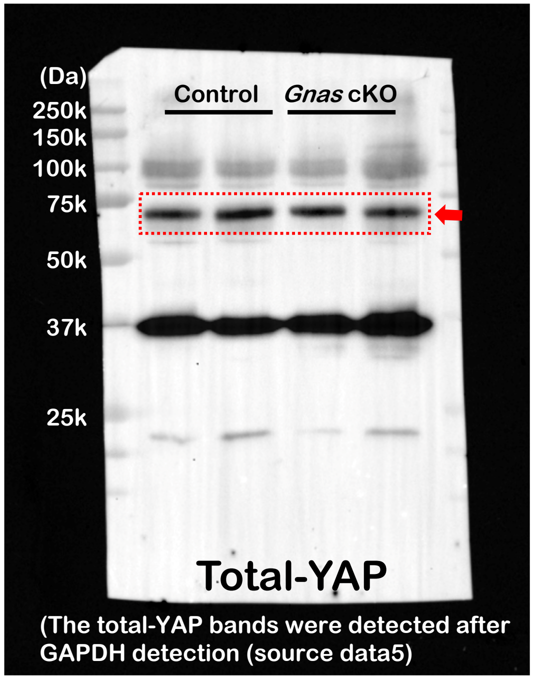

Figure5b-source data5

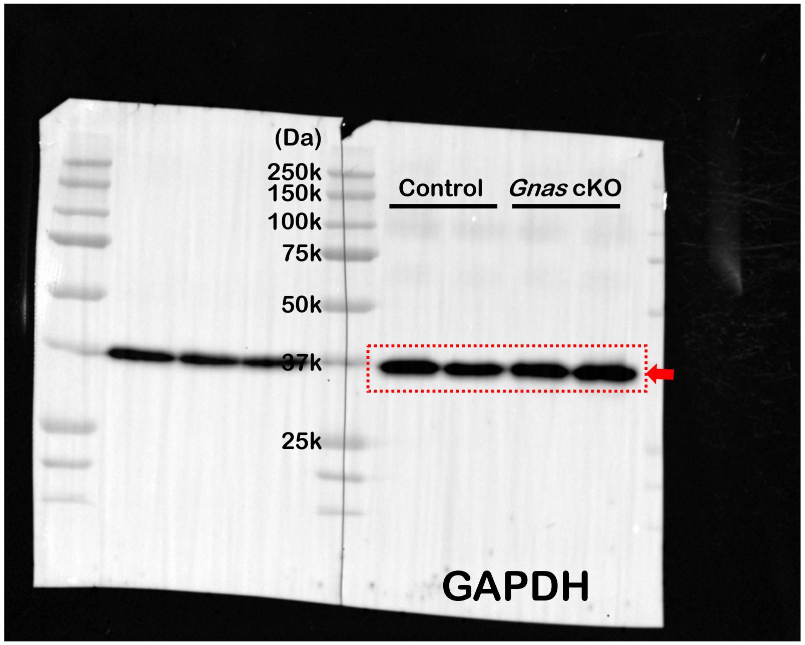

Figure5c-source data6

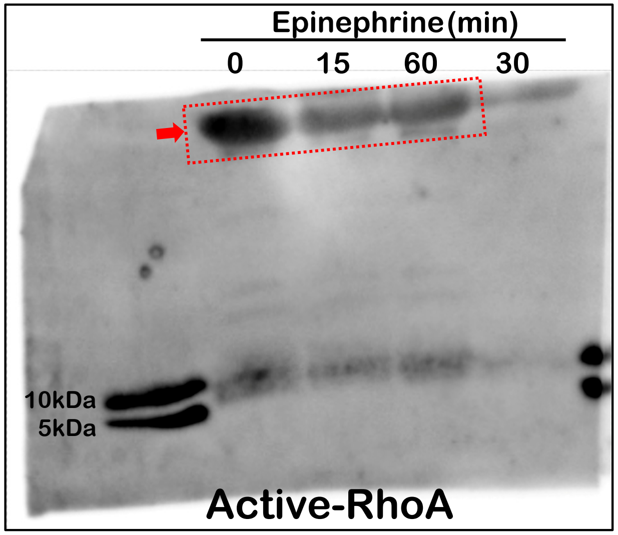

Figure5c-source data7

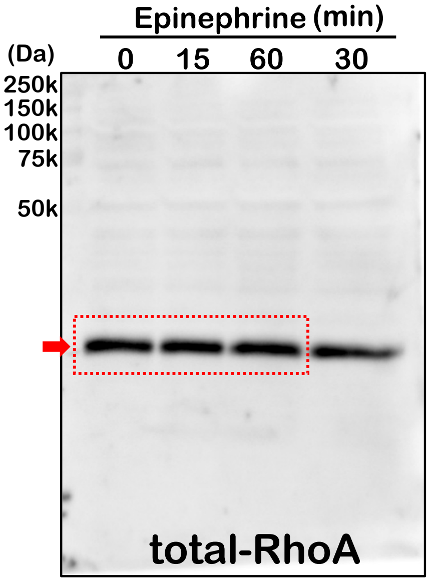

Figure5c-source data8

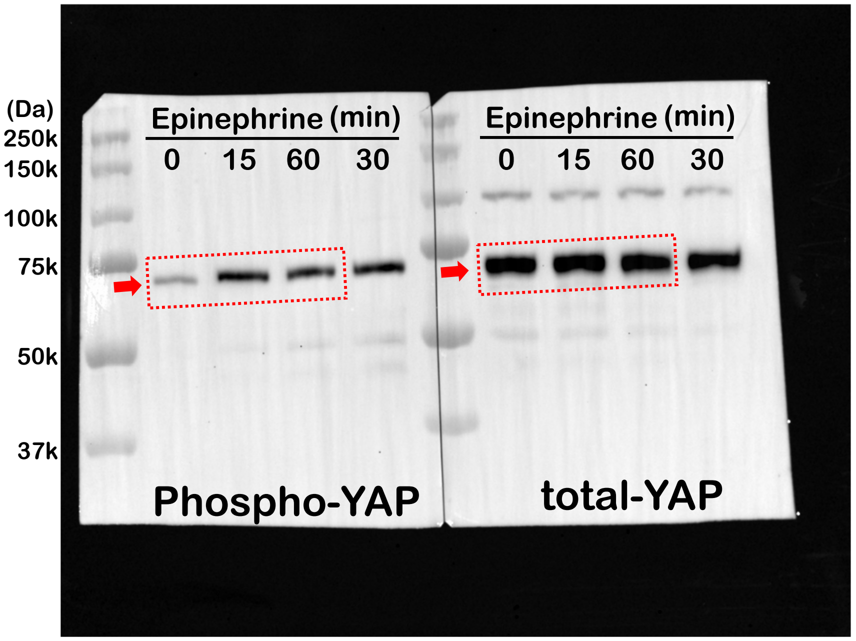

Figure5c-source data9

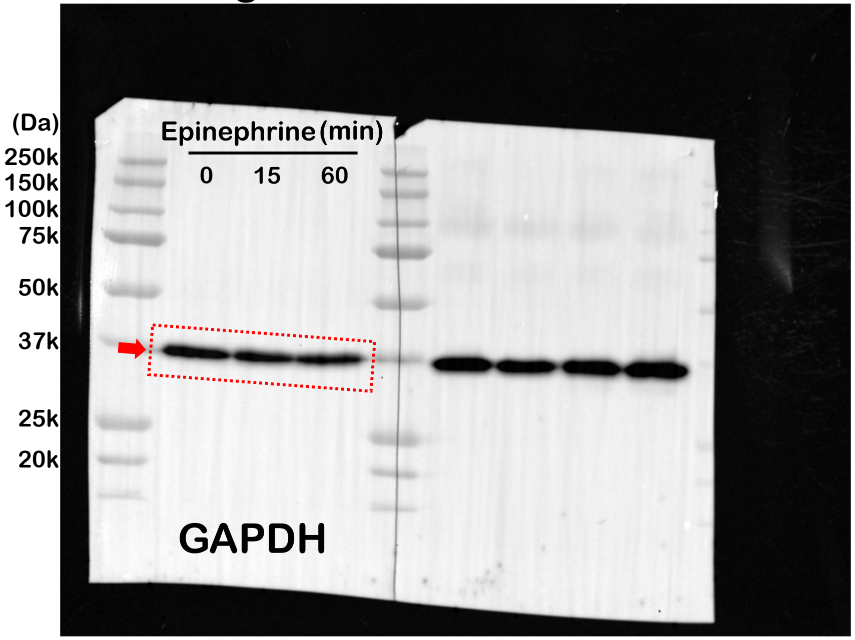

Supplement: Figure 5—source data 1. [file elife-74576-fig5-data1.zip › Source data Figure5/Figure5 source data.pdf]
