# Supplementary material for: Inhibition of β1-AR/Gαs signaling promotes cardiomyocyte proliferation in juvenile mice through activation of RhoA-YAP axis
Source: eLife. 2022 Dec 8;11:e74576. doi: 10.7554/eLife.74576 (PMC9767473; doi:10.7554/eLife.74576)
Supplement: Figure 5—figure supplement 1—source data 1. [file elife-74576-fig5-figsupp1-data1.zip › Source data Figure5-figure supplement1/Figure5-figure supplement1 source data.pdf]

Figure5-figure supplement1-source data1

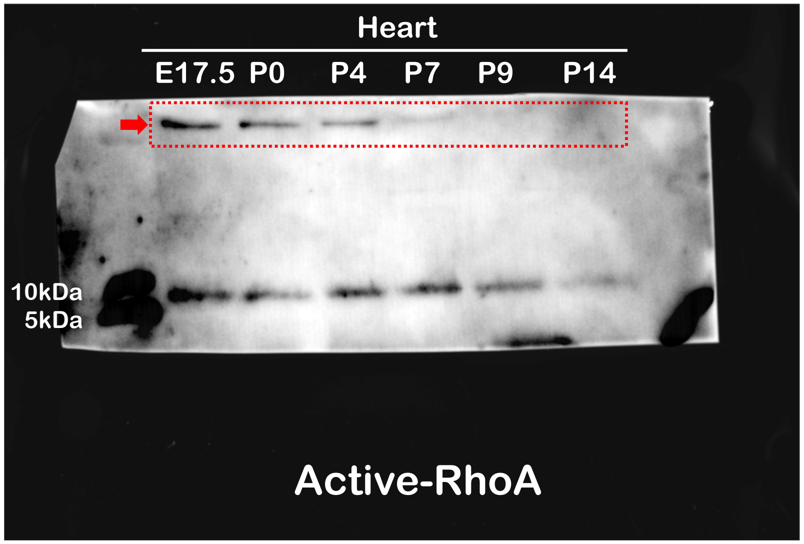

Figure5-figure supplement1-source data2

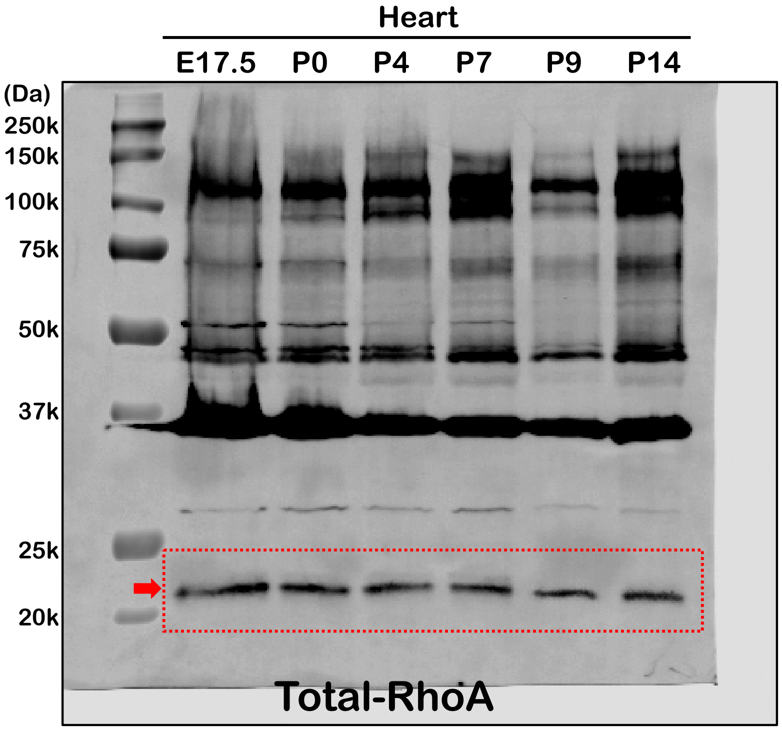

Since we reused the membrane several times, we saw extra bands that caused by previous antibodies. The total-RhoA antibody just detect RhoA, as shown in Figure5b source data2.
